# Tonga for Neuralgia

**Published:** 1886-07

**Authors:** 


### TONGA FOR NEURALGIA.

So many people are troubled with neuralgia that anything which tends to relieve it, even for a short time, is worth trying. One physician recommends tonga from actual experience in his own case. We quote what he says of that remedy, not only for neuralgia but other painful diseases :

"Tonga is the best medicine that I have ever given for neuralgia. It is safe, certain, pleasant to take, and no disagreeable or unpleasant symptoms—effects—follow its administration. My attention was called to it as a remedy for neuralgia about three years ago, and as I

had suffered intensely from neuralgia almost yearly for more than thirty years, I determined to try it upon myself. I mixed the fluid extract with an equal quantity of simple syrup, and immediately upon feeling the pain I took a large teaspoonful of the mixture, and repeated the dose every half hour, until four doses were taken, then I took a dose every hour until three doses were taken, and to my satisfaction the pain was held in check, did not become severe at all, as it had always done before. I repeated this course the next day and the next. The pain materially weakened the third day, and on the fourth it came not—I was well. I attended to my practice every day, and in the evening felt pleasant; indeed I felt so pleasant that I think the tonga must have had an exhilarating effect upon my nervous system. In all former attacks I was compelled to lie in bed for about one week, and was forced to take opium every day to mitigate the terrible pain. Since that time I have prescribed tonga in quite a number of cases of neuralgia with great success. I have also prescribed it in some cases, seemingly of a mixed character, some neuralgia, some rheumatism and some I don't know exactly what—perhaps the unknowable—with like success. When the pain is continuous, as it generally is in mixed cases, I give a dose every two hours during the day, and about twice during the night, and if relief is not obtained within two days, I increase the dose so that the patient will get about five or six drachms of the fluid extract in twenty-four hours. I esteem tonga far above all remedies known to me for neuralgia. The time was that the very thought of having the diseases was almost a terror to me, now I dread it not. Take tonga out of the medicine known as Tongaline, and what remains is not worthy of any consideration whatever. The first case of dysentery that I meet with I intend to try tonga, for I believe that it will prove of great utility in most cases of disease in which pain is a prominent feature. Its effects on the nervous system are certainly very peculiar and powerful, which demand for it careful investigation."

**Baron Liebig,** the great German chemist said: "We can prove with mathematical certainty that as much flour as can lie on the point of a table knife is more nutritious than eight quarts of the best Bavarian beer."

**A very Valuable Suggestion** is that which relates to rendering ball dresses and light gauzy summer garments uninflammable. If a little tungstate of sodium is added to the starching preparation, even thin clothes will be quite indifferent to flames. Here is a chance to save a number of serious accidents this summer.

